# Hoots and harm reduction: a qualitative study identifying gaps in overdose prevention among women who smoke drugs

**DOI:** 10.1186/s12954-021-00479-3

**Published:** 2021-03-07

**Authors:** Geoff Bardwell, Tamar Austin, Lisa Maher, Jade Boyd

**Affiliations:** 1 British Columbia Centre on Substance Use, 400-1045 Howe Street, Vancouver, BC V6Z 2A9 Canada; 2 Department of Medicine, University of British Columbia, St. Paul’s Hospital, 608-1081 Burrard Street, Vancouver, BC V6Z 1Y6 Canada; 3 Faculty of Medicine, Kirby Institute for Infection and Immunity, UNSW Sydney, Wallace Wurth Building, Sydney, NSW 2052 Australia

**Keywords:** Drug smoking, Harm reduction, Overdose prevention, Women-only spaces, Supervised inhalation services, Supervised consumption, Structural violence

## Abstract

**Background:**

Smoking or inhaling illicit drugs can lead to a variety of negative health outcomes, including overdose. However, most overdose prevention interventions, such as supervised consumption services (SCS), prohibit inhalation. In addition, women are underrepresented at SCS and are disproportionately impacted by socio-structural violence. This study examines women’s experiences smoking illicit drugs during an overdose epidemic, including their utilization of a women-only supervised inhalation site.

**Methods:**

Qualitative research methods included on-site ethnographic observation and semi-structured interviews with 32 participants purposively recruited from the women-only site. Data were coded and analyzed using NVivo 12 and thematic analysis was informed by gendered and socio-structural understandings of violence.

**Results:**

Participants had preferences for smoking drugs and these were shaped by their limited income, inability to inject, and perceptions of overdose risk. Participants expressed the need for services that attend to women’s specific experiences of gendered, race-based, and structural violence faced within and outside mixed-gender social service settings. Results indicate a need for sanctioned spaces that recognize polysubstance use and drug smoking, accommodated by the women-only SCS. The smoking environment further fostered a sociability where participants could engage in perceived harm reduction through sharing drugs with other women/those in need and were able to respond in the event of an overdose.

**Conclusions:**

Findings demonstrate the ways in which gendered social and structural environments shape women’s daily experiences using drugs and the need for culturally appropriate interventions that recognize diverse modes of consumption while attending to overdose and violence. Women-only smoking spaces can provide temporary reprieve from some socio-structural harms and build collective capacity to practice harm reduction strategies, including overdose prevention. Women-specific SCS with attention to polysubstance use are needed as well as continued efforts to address the socio-structural harms experienced by women who smoke illicit drugs.

## Background

It is well established in critical drug policy research that social, structural, and physical environments affect the health and wellbeing of marginalized communities [1–8]. There is a pressing need to implement interventions that address drivers of drug-related harms (e.g., gender and race-based violence, drug criminalization, lack of harm reduction services). Supervised consumption services (SCS) are one such intervention whereby people can access sterile injecting equipment and use pre-obtained drugs in a legally sanctioned environment under the supervision of staff trained to respond in the event of an overdose [9]. Presently, there are over a hundred SCS internationally [10], including stand-alone sites [11, 12] and integrated models within healthcare facilities [13, 14]. However, despite the fact that women who use drugs are disproportionately impacted by socio-structural violence compared to men [15, 16], few SCS are women-only [17, 18]. Further, while there are numerous SCS in Europe that provide supervised inhalation services [19], most SCS in North America do not provide this service [20], even though people who smoke drugs are also at risk of overdose and drug related-harms [21, 22]. Research on unsanctioned inhalation sites in Canada demonstrates how these services are not only cost-effective [23], but also improve health and safety measures and protect clients from violence, policing, and stigma [24, 25].

Drug inhalation can be a safer method compared to injecting, with some studies suggesting lower rates of drug dependence, lessened risk of infectious disease transmission, decreases in overdose risk (in some settings), and lower rates of co-occurring physical and psychological problems [26–29]. Some people who use drugs (PWUD) prefer smoking compared to other methods of consumption (e.g., injecting, swallowing) [30]. For example, a Canadian study among people who use stimulants indicates that some people prefer smoking (rather than injecting) crack cocaine due to its perceived efficiency, ease of ingestion, and cost [31]. A recent systematic review on smoking heroin (i.e., diacetylmorphine) also identifies smoking as providing a greater ease of administration among PWUD [32], though this can be complicated by drug supply composition and toxicity [33, 34]. Other research indicates that smoking, rather than injecting, heroin as a mode of consumption is the more common cultural practice among some racialized communities [35]. While there are a variety of reasons why some PWUD smoke drugs, there are a host of health-related harms associated with this practice such as leukoencephalopathy [36] and overdose [37, 38]. Among women who smoke crack, high-frequency crack use (at least 5x/day) has been associated with human immunodeficiency virus-related sexual risk activities (e.g., unprotected oral sex) [39]. In one study in Vancouver, hepatitis C virus prevalence was high among people of all genders who used crack with no history of injection (43%), but for women who smoked crack, prevalence was significantly higher at 58% [40]. Women sometimes lack their own equipment for safer use and are consequently “second on the pipe,” which increases their risk to communicable infections [41], including the novel coronavirus diseases 2019 that emerged after study completion. Furthermore, for those who use methamphetamine, one study found that women were less likely to inject methamphetamine compared to men and more likely to report other methods (e.g., smoking, snorting) [42]. Additionally, a supervised inhalation services feasibility study found that among people who smoked crack cocaine, women reported greater willingness to utilize these services [43].

Gendered and socio-structural understandings of violence provide a useful conceptual lens for examining the experiences of women who smoke illegal drugs. Violence is institutionalized by structural drivers, including inequality, systemic racism, and discrimination, which have lasting impacts on the health of vulnerable populations [44]. Critical researchers have emphasized the need to move away from neoliberal approaches to marginalization and violence that individualize these experiences [45, 46] toward examining how experiences of social suffering are connected to larger social and structural power relations [47]. Street-entrenched individuals are often blamed for their experiences of poverty and social suffering despite the fact that these everyday acts of violence are condoned and perpetuated by macrolevel contexts such as capitalism, white supremacy, and patriarchy [45]. Numerous studies on women who use drugs have identified the need for public health interventions, including overdose prevention measures, that address the ways in which gendered social and structural violence impacts women’s overall health and wellbeing [16, 45, 48–53].

Communities across North America are experiencing an ongoing overdose epidemic that has largely been fueled by the increased presence of illicitly manufactured fentanyl contaminating the drug supply [54–57]. Recent data from the Canadian province of British Columbia (BC) indicates that women accounted for 20% of overdose deaths in 2020 [58], and that Indigenous women are disproportionately affected [59], with an overdose mortality rate for First Nations women at least 8.7 times the rate for non-Indigenous women in 2019 [60]. Additionally, BC coronial data indicates that, since 2017, smoking has been the most common mode of drug consumption among decedents, accounting for 40.1% of overdose deaths in 2019 [37]. Between 2016 and 2017 the majority of women’s overdose deaths were not via injection; 31% involved inhalation (and the remaining involved intranasal and oral at 31% and 11% respectively) [61]. Harm reduction programing in North America is largely approached through a gender-neutral lens, which limits the capacity of some services in providing gender-specific targeted interventions [16, 62–65]. Indeed, these data demonstrate the urgency for gendered, culturally attentive, and inhalation-specific public health interventions to prevent overdose and other drug-related harms.

Only two women-only (gender fluid and transgender inclusive) SCS exist within the epicenter of Canada’s overdose crisis in the Greater Vancouver Area of BC. One, located in Vancouver’s inner city, targeted women who inject drugs during the study period and did not provide supervised inhalation services [18]. The other SCS, the focus of this study, is located within a transitional housing and drop-in service in the city of Surrey and has a smoking area on its premises. Surrey is the second most populous municipality within the Greater Vancouver Area, with a population of 517, 885 [66]. Since 2010, Surrey has consistently recorded the second highest rate of overdose deaths in the province (next to Vancouver), with 119 deaths in 2019 [58]. While Surrey has a variety of health and social services that target PWUD, including SCS and needle and syringe programs, there is minimal women-specific harm reduction programming. Furthermore, harm reduction interventions during the current overdose crisis have largely focused on opioid use, while less attention has been paid to stimulant and polysubstance use. We sought to examine women’s perceptions and practices of harm reduction while utilizing a novel women-only SCS in Surrey that accommodates smoking and polysubstance use.

## Methods

### Study setting

**Fig. 1:**
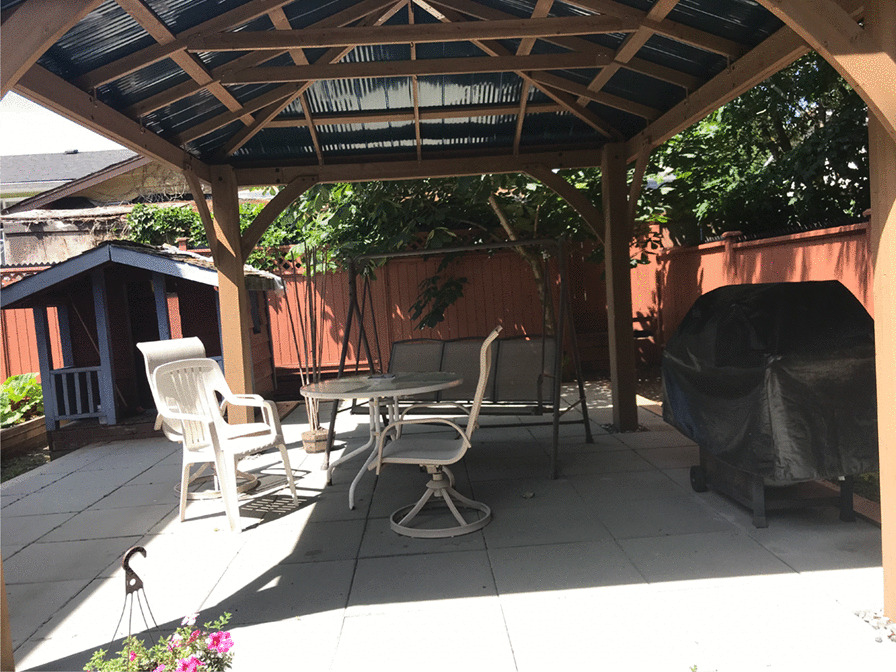
Women-only smoking space

This study was conducted at a women-only transitional housing and drop-in service in Surrey, Canada, located approximately 34 km away from Vancouver’s inner city. The transitional housing building is a small two-story house located in a residential area close to a shopping center and public transit access and is managed by a non-profit organization that primarily focuses on ending violence against women. It has two designated drug use rooms on each floor where women can consume drugs, though smoking is prohibited in these indoor spaces. One of the harm reduction rooms is in the drop-in area where women can access educational materials and drug-related equipment (e.g., pipes, needles, syringes), a washroom, and an informal setting with a kitchen and couch area for sleeping or socializing. The kitchen leads to a secluded backyard with a grassy area and a covered patio for smoking both legal and illegal substances (see Fig. 1). Support staff are on site at all times, and while the consumption spaces (both indoor and outdoor) are not consistently supervised, they are checked on regularly by staff. The organization also partners with Indigenous Elders and has provided some Indigenous-specific programming.

### Data collection

Data were collected from June to September 2019 via qualitative and rapid ethnographic research methods. Qualitative research [67] provides useful tools for gaining a broader understanding of the subjective experiences of women who smoke drugs. This approach allows researchers to feature participants’ experiences through interview quotations, which are a means to further “give voice” to marginalized communities [68, 69]. Additionally, unlike traditional ethnography, which is contingent on researchers immersing themselves within a community for a long period of time, rapid ethnography relies on a research team’s familiarity with the subject matter (e.g., drug use, harm reduction) and context-specific issues (e.g., women’s drug use, overdose epidemic) to more quickly produce data on local community understandings [70].

Qualitative semi-structured interviews were conducted with 32 women who accessed drop-in and/or transitional housing services at the study site. Participants were purposively recruited with the assistance of staff and peer workers and provided written informed consent. An interview guide was designed in consultation with a community advisory board comprised of women with lived experience of drug use and/or homelessness. The guide focused on experiences of drug use and homelessness, drug consumption methods, barriers to accessing local health and social services, and service utilization at the women-only site. Confidential interviews were conducted in a private room on-site by TA and JB. Interviews were approximately 45 to 60 min in length. Each participant received a $30 (CAD) cash honorarium. Interviews were audio-recorded and transcribed by a professional transcription service. All identifying information was removed from the transcripts to protect confidentiality, and each participant was assigned a participant number as an identifier.

Interviews were accompanied by approximately 70 h of on-site ethnographic fieldwork conducted by TA. This included overt participant observation and informal conversations in order to witness service access and better understand the contextual factors that affected service utilization, including drug use and harm reduction practices. Additionally, photographs of the physical setting were taken to contextualize the study setting through visual images [71, 72]. These ethnographic methods were further used to enhance the validity of the interview data and assist with data triangulation [73, 74]. Ethical approval for the study was obtained through the University of British Columbia/Providence Health Care Research Ethics Board.

### Data analysis

Multiple members of the research team individually reviewed portions of the interview transcripts and fieldnotes to develop a list of potential themes and subthemes for thematic analysis. We then met as a group to discuss and develop a coding framework. Codes were developed using themes that emerged from the data as well as a priori themes [75]. We utilized qualitative data management software (i.e., NVivo 12) to organize and code the data prior to analysis [76]. Data analysis was further informed by theoretical approaches to socio-structural violence (as described in the Introduction) as well as a feminist and gendered lens to develop our analysis beyond descriptive themes.

### Participants

All 32 participants were cis-gender women ranging in age from 22 to 55 years. Fourteen participants identified as Indigenous, 15 as white, 3 as Black, and one as South Asian, with some participants identifying as multiple categories. All but three women smoked drugs and nine had experienced at least one overdose in the last year. The majority of participants were polysubstance users, sometimes using substances concurrently (therefore, unless otherwise noted, when participants described their substance use, they did not always identify specific drugs). See Table 1 for further demographic characteristics.

**Table 1:** Sample characteristics (n = 32)

**Age**	
Average (median)	40
**Homeless (past year)**	
Yes	26
No	6
**Income generation (past 30 days)**	
Social assistance	29
Sex work	15
Theft	11
Drug selling	9
Recycling	6
Stipends from volunteering	5
Part-time employment	3
Other	5
**Substance use (past 30 days)**	
Crystal methamphetamine	26
Heroin	20
Fentanyl	19
Crack cocaine	12
Alcohol	9
Cocaine	7
Speedball	5
Prescription opioids (extra-medical)	4
Other	4
**Drug consumption method**	
Smoke	29
Inject	12
Snort	8
Swallow	5
**Frequency of drug use**	
Daily	28
Less than daily	4
**Overdose experience (past year)**	
1 overdose	3
2 overdoses	1
3 or more overdoses	6

## Results

### Context: Socio-structural violence

Participants’ descriptions of their day-to-day experiences of violence, discrimination, drug use, and health and social service navigation, outside of the women-only site, revealed their structural vulnerability. The majority of participants (n = 26) had experienced homelessness in the past year. Many described their experiences of being precariously housed with limited options that included “flop houses” (i.e., inexpensive lodging), sleeping on the streets, or accessing emergency shelters. Participants described fleeing from abusive relationships, only to also experience gender-based violence and harassment when homeless. Participants recounted these experiences within street-based drug scenes, at drop-in spaces, and within mixed-gender shelter spaces. For example, one woman stated: “I got sexually assaulted in the shelter I was living in…so I had to move out of there, and then my ex was there, and we were fighting again. I needed to be away from him for a while” (P7, mid 40 s, Indigenous).

Participants described experiences of intersecting discrimination. While some emphasized positive experiences accessing Indigenous-specific services, they also described experiences of racism in other settings including in housing, public locations, and in relation to policing. For example, one participant stated: “The way [the police] respond to Aboriginal women that’s been beat up versus how a white woman is taken in, it is just different. It’s racism alive and well there” (P17, mid 50 s, Indigenous). Participants also reported experiences of discrimination when accessing a variety of services, disclosing being treated “like animals” due to their involvement in drug use and sex work. Participants noted that rules at shelters and other support spaces that prohibited drug use, including inhalation, led them to leave these service settings to smoke elsewhere, primarily on the street, in public washrooms, or alleyways. These experiences were described as unsanitary, for example, one woman stated, “I had to go down the alleyway and stand in a doorway where people defecate. And so like and that’s not cool for me” (P1, mid 30 s, white). Furthermore, this displacement required women to consume drugs in potentially less safe environments, hidden away from support staff. Participants emphasized the need for not only safer drug use spaces, but also harm reduction services that specifically target women, including women-only spaces. As expressed by one participant:

Women do need their own space to heal and to be able to build a support system because a lot of women like myself are vulnerable and don’t really trust men and would sooner just sit back and say nothing if it’s a man sitting across from them. (P17, mid 50 s, Indigenous).

All participants expressed the need for services that allow for drug use, including smoking, and that attend to women’s specific experiences of gendered, race-based, and structural violence. The women-only site was described as integral to providing women-attentive services.

### Framing preferences for smoking

Participants identified diverse methods of drug consumption, including injecting (n = 12), snorting (n = 8), and swallowing (n = 5); however, smoking was most common (n = 29) and the majority of participants discussed having a preference for smoking drugs. Preferences were framed by negative views on other methods of consumption (i.e., injection), the longevity of the high when smoking, poor venous access, and limited resources (allowing participants to “stretch” their use with smaller quantities when smoking). Some participants described a dislike for, or fear of, needles, and described the idea of injecting drugs as making them “squeamish,” “afraid,” or feeling “extreme discomfort.” For example:

I’m not a needle thing. I don’t like them. I think they’re ew, they’re just gross. I couldn’t put needles in my arms. I’ve been 20 some years of using, I’ve never ever touched a needle…It’s just scary. (P3, early 40 s, white).

Many participants explained that their general fear of needles led them to choose smoking drugs as a route of administration.

Participants who had a history of injection drug use described experiencing different sensations from smoking versus injecting. Smoking was described as lasting longer, as seen in the following excerpts from participants who smoke heroin and/or fentanyl:

Smoking it I seem to find it lasts longer and you have a way better high. Whereas when you smash (i.e., inject) it it’s like, bang you’re high and then it doesn’t last that long. (P33, late 30 s, Indigenous).

I don’t inject very much anymore. I just smoke, yeah. [I: Why is that?] My veins are getting old and tired. [I: Okay, so it’s easier to do the smoking?] Yeah. [I: Does it have the same effect?] I like it better actually. [I: Why?] It just lasts longer, it seems. (P2, early 40 s, white).

Not only does the latter quote describe the longevity of the high for heroin/fentanyl compared to injecting, but also how decisions to consume drugs via other methods such as smoking are further framed by health-related reasons that limit the ability to inject (e.g., collapsed veins).

Given the longevity of the high for some women, the decision to smoke rather than inject was also shaped by participants’ limited resources as well as the drug quality. According to one participant who primarily uses heroin/fentanyl:

I smoke mostly, inject if it’s necessary. Like depends on how much I have, right, if I only have 20 bucks worth then I’ll smoke half and then inject the half. [I: What makes you choose one over the other?] Depends on the quality I guess…because if it’s garbage then you’re just gonna want to inject it all because I mean might as well get everything in…but if it’s good stuff then I’ll smoke it. (P25, early 30 s, Indigenous).

Thus, economic factors shaped participants’ preferences for smoking drugs.

### Smoking, overdose risk, and perceived harm reduction

Some participants perceived smoking drugs to be a harm reduction practice and described it as a safe way to use drugs, as illustrated in the following quote: “I smoke [heroin/fentanyl] on tinfoil. I don’t sniff anymore and I don’t [inject]. If you have to get it in your system because you’re sick…I find that [smoking is] the safest way” (P1, mid 30 s, white). Some participants also explained that they perceived injection drug use as more dangerous: “You overdose if you use syringes” (P18, late 30 s, white), and “Every time I fixed, I OD’d and it’s not fun…[I: Has ODing ever changed the way you use drugs?] Yea. I just smoke it now” (P21, late 40 s, white). Smoking was perceived by some as a safer practice than injecting, particularly as it related to overdose risk.

Other participants, however, described concerns about potentially overdosing from smoking drugs, particularly in the context of an overdose crisis and how this impacted their smoking practices. One woman who smokes both crystal methamphetamine and heroin/fentanyl (and sometimes concurrently), spoke about hiding her drug use from her partner. When asked about her overdose risk when hiding and using alone, she said: “I’m careful…like as long as I smoke it, but there’s a risk, I guess so. I’m pretty sure there is” (P3, early 40 s, white). This participant later reported an experience overdosing from smoking, so while she described smoking as safer than injecting, she also identified potential risks. Another participant spoke about overdose risk from smoking stimulants. She said: “I heard you could overdose even smoking crack, which surprised me. But it’s in my head now, right, I mean you can OD but I never did big hits anyway” (P14, early 50 s, Indigenous). Both of these participants identify the potential risk of overdosing while smoking drugs but also describe using caution and not using large quantities in order to mitigate risk.

When participants were asked how the overdose epidemic generally, or witnessing their peers overdose specifically, affected their drug use, many described how they had adapted their use by smoking smaller amounts more frequently as an overdose prevention practice. According to one participant: “You take a hoot or whatever, smoke it and see how it goes…and usually by the second or third hoot, you know” (P28, early 20 s, Indigenous). After seeing others overdose, another participant described her change in practice: “I think I just take smaller hoots now…just in case that would happen to me” (P8, early 40 s, white). Finally, another participant suggested that the overdose crisis not only affected how much she used but also the location of use: “It makes me more conscious of where I use and how much I use” (P17, mid 50 s, Indigenous). These quotes demonstrate not only the ways in which participants navigated risk when smoking drugs and adapted harm reduction practices, but also point to the need for safer spaces for drug inhalation and other strategies to mitigate overdose risk.

### Sociability and drug sharing

Having a sanctioned smoking space at the women-only transitional housing and drop-in center was described as a much-needed harm reduction intervention among participants. In addition to participants’ expressed benefits of a smoking-specific service, they noted how this informal smoking area enabled socializing with other women, which was demonstrated through their descriptions of the practice of drug sharing. Sharing a variety of drugs was described as commonplace particularly for those who are smoking. For example: “I usually snort (crystal methamphetamine), but when I’m around a crowd, we smoke it” (P31, mid 50 s, white). Other participants described sharing drugs as a way to support someone in need (e.g., to help someone avoid withdrawal symptoms) as illustrated in the following quotes: “You do have a group of people who will help you out when you don’t have something” (P28, early 20 s, Indigenous), and “Outside I would share with some of the girls in here, you know, if they don’t have any drugs” (P14, early 50 s, Indigenous). One participant, when asked about her thoughts on the designated smoking area, not only described the social aspect of sharing drugs but also described the group setting as an overdose prevention measure. She said: “It saves women from being out there by themselves and having a chance of overdosing, and it gives a woman somewhere to sit and get along with each other” (P25, early 30 s, Indigenous). Sociability matters in the context of drug use and can also play a role in overdose prevention whereby peers can supervise consumption, administer naloxone, and call for help. However, some participants were also wary about sharing drugs at the site, noting instances where they had been taken advantage of or had concerns about potential theft. For example, “I feel like they’re gonna steal my drugs. When I have crack I’m just like super paranoid of the person sitting beside me” (P12, early 40 s, Indigenous). Given their structural vulnerability, it is unsurprising that some women expressed such concerns. Despite these concerns, the women-only sanctioned inhalation site provided a safer space where women had the ability to collectively enact harm reduction strategies via sociability.

## Discussion

In summary, participants noted that the lack of sanctioned smoking spaces within social services led them to use in public spaces which increased the risk of overdose and gendered and racialized violence. Multiple participants described preferences for smoking drugs that were shaped by their injecting ability, limited income, and perceptions of overdose risk. Given these preferences, participants discussed a need for designated smoking spaces such as the women-only SCS which accommodated a range of drug consumption practices, and served to further enable a sociability among women, including the sharing of drugs with others in need and the ability to respond in the event of an overdose.

Our findings also highlight the ways in which social, structural, and physical environments impact the experiences of women who smoke drugs. Participants described persistent violence, discrimination, and harassment on the street as well as within multiple social services in Surrey and other parts of the Greater Vancouver Area. Our findings regarding participants’ experiences of socio-structural violence outside of the women-only site are consistent with studies among structurally vulnerable women who use drugs [40, 48, 50, 52, 77]. For example, previous research has illuminated how socio-structural contexts can negatively impact women’s health, shaping experiences of poverty, racism, and sexism in the inner city, and how crack was used in response to the everyday violence that women experienced (e.g. to stay awake to avoid assault) given their limited health management opportunities [48]. A lack of women-specific harm reduction responses including women-specific and culturally appropriate healthcare for Indigenous women, also shape women’s drug use [48]. Participants in our study described their experiences of race- and gender-based discrimination and violence within both street-based drug scenes and at mixed-gender drop-in and shelter services. Due to their gendered experiences of drug use and accessing services, and against the backdrop of an ongoing overdose crisis, participants described a need for safer spaces to use drugs, and specifically ones that allow women to smoke drugs.

Research on low-barrier SCS has demonstrated how these spaces are gendered and can reproduce existent unequal power relations between men and women that can engender social violence thereby potentially jeopardizing some women’s access to these life-saving services [50]. A recent study of a low-barrier SCS in Toronto that allowed on-site smoking, for example, identified the sexual harassment of women by men within their smoking space [24]. While multiple studies have recommended the implementation of supervised inhalation services [20–22, 43, 78, 79] to address safety concerns and the experiences of racism and everyday violence among women who smoke drugs, there is also a need for culturally attentive women-only spaces [18, 50, 77], including non-medicalized peer-led SCS models [80], targeting Indigenous and other racialized women who continue to experience racial and stigma-based barriers to health services [81]. Research on a women-only SCS in Germany found that 80% of participants felt more safe among women compared to mixed-gender services [17], further emphasizing the need for gender-specific services for women who use drugs.

The meaning of safety within women-only spaces is not just about being *safe from* gendered violence, but also about being *safe to* enact agency [82]. Past research examining safer crack use among women has illustrated women’s agency and capacity to care for themselves and others via harm reduction practices; however, this study also reported how structural violence constrains this agency and therefore demonstrates a need for safer spaces for women to smoke drugs and enact risk reduction strategies [77]. The women-only site provided women opportunities for choice (e.g., control over their drug use, drug consumption methods) and support via drug-sharing and sociability. A recent study of peer-led informal harm reduction services located within communal drug use spaces in Toronto illustrated how PWUD provide care for others in these spaces through acts such as drug-sharing and overdose response [83]. Drug-sharing is considered a criminal offence in Canada and elsewhere (i.e., trafficking) and is thus a prohibited practice in federally sanctioned SCS [84, 85]; however, in these communal spaces sharing was described an act of “mutual aid” [83]. A study of a SCS in Germany describes how enforcing rules that prohibit sharing can also increase clients’ exposure to police interventions if they are unable to share on site [85]. In our study setting, given the economic realities of women living in poverty, sharing drugs was seen as a way to help other women who did not have any drugs and/or were experiencing withdrawal symptoms. Past research among structurally vulnerable PWUD has highlighted how, via sharing, PWUD incur moral and economic debts within their social circles with the expectation of future reciprocal exchanges of drugs [86]. Furthermore, given that the prohibition of sharing drugs was not overtly enforced at the women-only space, the site provided additional protection from some of the effects of criminalization (e.g., drug confiscation, arrest) if women otherwise had leave the premises to share. Past studies have identified safety, immunity from arrest, and avoiding police encounters as additional motivations for utilizing SCS [8, 87, 88].

The sociability among our study participants also provided an informal overdose response network of other women who smoke drugs. Research among people who smoke crack has emphasized how being in a safe space among trusted friends alleviates the risks associated with public use and using alone [3]. Given that the majority of overdose deaths in BC occur indoors and are often the result of people using toxic drugs alone [89], sanctioned shared (i.e., peer-to-peer) and supervised smoking sites, particularly those with gender-specific and culturally attentive programing, have the potential to mitigate drug-related risks and harms (e.g., violence, overdose) impacting women who smoke drugs.

Some participants expressed concerns about overdose risk when smoking, despite their limited abilities to mitigate such risk in other formal service settings (e.g., mixed-gender shelter and drop-in services). In the Canadian context, many women-specific housing and shelter services, including transitional houses and violence against women shelters, have policies prohibiting drug use and are often unequipped to support women who use drugs [90], illustrating a gap in current policy and practice. Other participants specifically reported smoking, rather than injecting, drugs in an effort to mitigate overdose risk. While smoking can be a harm reduction measure [27, 29, 91], the toxicity of the illicit drug supply in North America is high [58, 92], and smoking these toxic substances continues to lead to a significant number of fatal overdoses in BC [37]. The overdose concerns identified by participants, coupled with drug toxicity and inhalation overdose mortality data, illuminate the clear need for the scale up of inhalation services in a range of environments in order to address this significant gap in public health overdose prevention interventions.

There are some limitations to this study. While we recruited a diverse sample, participant views may not be representative of all the women who accessed services and/or the SCS. Participants were recruited directly from the women-only site, so the experiences of women who smoke drugs in Surrey who do not access this site are not represented here. Lastly, while this women-only site was transgender-inclusive, all participants were ciswomen and our findings therefore may not be applicable to transgender women. Future research on drug use practices should be prioritized to examine the experiences of structurally vulnerable transgender and gender-fluid people, particularly given the well-documented negative experiences this population face in emergency shelters, transitional housing, and other gendered spaces [93–95].

In conclusion, our findings highlight current policy and programmatic gaps across most health and social services for people who use drugs, particularly as they relate to the intersections of drug inhalation, polysubstance use, as well as structural, gendered-, and race-based violence. Given the high overdose risk among people who smoke drugs in our study setting, inhalation services are urgently needed. The women-only smoking space provided temporary reprieve from some socio-structural harms and enabled participants to practice harm reduction strategies, including overdose prevention. However, there is a need for the scale-up of women-specific culturally attentive harm reduction interventions across multiple jurisdictions that recognize diverse modes of drug consumption in order to ensure equitable access and improve harm reduction service provision, as well as health outcomes, among women who smoke drugs.

## Data Availability

The datasets are not available due to the sensitive nature of the study, as they contain confidential information that could compromise participant confidentiality and consent.

## Abbreviations

BC: British Columbia
PWUD: People who use drugs
SCS: Supervised consumption services
